# DARC-NESS: a mastery-based cognitive-behavioral model for treating chronic nightmares in youth

**DOI:** 10.3389/frsle.2026.1772987

**Published:** 2026-02-27

**Authors:** Lisa DeMarni Cromer, Emily Kaier Cromwell, Lauren E. Prince, Tara R. Buck

**Affiliations:** 1 Department of Psychology, The University of Tulsa, Tulsa, OK, United States; 2 Department of Psychiatry, Child and Adolescent Services, University of Rochester Medical Center, Rochester, NY, United States; 3 Department of Psychiatry, The University of Oklahoma School of Community Medicine, Tulsa, OK, United States

**Keywords:** cognitive behavioral therapy for youth, nightmare disorder, nightmare maintenance, pediatric sleep, self-efficacy

## Abstract

Theories of chronic nightmare maintenance highlight dysfunctional beliefs about sleep and nightmares, distress and arousal, anticipatory anxiety, maladaptive sleep habits, and sleep deprivation as perpetuating factors that maintain nightmare disorder over time. Theories of nightmare treatment suggest that self-efficacy is a common factor in nightmare mitigation. The current article introduces DARC-NESS, a multi-component theory of nightmare maintenance that emphasizes nightmare self-efficacy as a central mechanism influencing the maintenance cycle at multiple points. DARC-NESS is a mnemonic for the model's components: Dream (nightmare) content, Appraisals, Resources for regulation, Conditioned arousal, Nightmare Efficacy, Sleep hygiene and patterns, and Sleep quality and quantity, that interact to perpetuate nightmares. This model provides the theoretical basis for cognitive behavioral therapy (CBT) for child nightmares. The manuscript proposes treatment counterparts to each model component and presents a case illustration demonstrating how these interventions can disrupt the vicious cycle of chronic nightmares. Finally, flexible clinical applications are offered to guide clinicians in selecting and sequencing modular intervention elements to match individual case presentations.

## 1 Introduction

Sleep quantity and quality are essential for emotional, cognitive, and physical development, yet a frequently overlooked barrier to restorative sleep in childhood and adolescence is chronic nightmares. Chronic nightmares can be responsive to treatments, but when they persist, cause sleep fragmentation, disrupt sleep continuity and total sleep time, and elevate pre-sleep arousal, all of which undermine daytime functioning and overall well-being (Gauchat et al., 2020). Although occasional nightmares are common and typically benign, persistent and distressing nightmares represent an understudied malleable contributor to poor sleep health across development (Cromer et al., 2022; El Sabbagh et al., 2023) and may amplify risk for broader mental health problems (Gieselmann et al., 2019; Gill et al., 2023). As the field advances educational and cognitive-behavioral interventions to promote sleep, chronic nightmares remain insufficiently integrated into models of sleep quality and intervention, despite being modifiable. There is considerable evidence that nightmare treatments are effective in adults (Gieselmann et al., 2019). The current manuscript presents the theoretical basis for a brief evidence-based treatment for youth (Cromer et al., 2024). In a recent systematic review, Gill et al. (2023) expressed an “urgent need” (p. 280) for rigorously conducted clinical trials targeting nightmares in youth. This gap underscores the importance of a strong theoretical framework to guide intervention development. In line with this need, the current manuscript presents a nightmare maintenance model (i.e., factors that sustain nightmares over time) that identifies nightmare efficacy as a central, developmentally relevant mechanism. Nightmare efficacy refers to the belief in one's ability to control, cope with, or reduce nightmares, whereas mastery reflects the accumulation of successful experiences using skills to positively impact nightmares and sleep. Thus, efficacy represents a central cognitive mechanism that supports engagement in skill-based practice and the development of mastery over time. The model has been refined through several pilot studies and a clinical trial demonstrating promising reductions in nightmares and improvements in mental health (Cromer et al., 2024). These findings serve as the theoretical foundation for transdiagnostic treatment for improving nightmares and sleep in youth ages 6–17 years. By articulating the model and illustrating its clinical application, this manuscript aims to support clinical applications in the short term as well as the next phase of research.

## 2 Clinical features and diagnostic distinctions of chronic nightmares

Nightmares can be symptomatic of other mental health diagnoses, but when they cause sufficient distress and impairment, they may be independently diagnosed and treated. The Diagnostic and Statistical Manual of Mental Disorders, Fifth Edition, Text Revision (DSM-5-TR) definition of nightmare disorder includes, “repeated occurrences of extended, extremely dysphoric, and well-remembered dreams that usually involve efforts to avoid threats to survival, security or physical integrity” (American Psychiatric Association, 2022, p. 457). A defining feature of a nightmare is that an individual becomes rapidly alert upon waking and has significant distress and/or functional impairment. Modifiers are mild (<1 per week), moderate (one or more per week) and severe (nightly; American Psychiatric Association, 2022).

Chronic nightmares have deleterious effects on mental and physical health (American Psychiatric Association, 2022, p. 460) and nightmare treatment can improve other threat-related disorders and possibly depression (Sheaves et al., 2023). Further, children's nightmares can disrupt household members' sleep (Li et al., 2011). Although sleep health has gained increasing attention in recent decades, nightmares, particularly in youth, remain understudied, underdiagnosed and rarely treated (Cromer et al., 2022; Nadorff et al., 2015). Nightmares are often conceptualized as secondary symptoms of other mental health conditions [e.g., posttraumatic stress disorder (PTSD)] despite evidence that standalone treatments are effective (Gieselmann et al., 2019; Gill et al., 2023).

Differential diagnosis is essential for treatment planning because most conditions that mimic nightmares will not respond to a unified protocol for nightmare treatment. Diagnosis relies on clinical evaluation involving the child and caregiver that considers the child's recall of the episode, the timing of the event, and behavioral observations. Below, we highlight common differential diagnoses relevant to clinical assessment.

Nightmares should be distinguished from non-rapid eye movement (NREM) sleep arousal disorders, such as sleep terrors (Leung et al., 2020). NREM disorders of arousal occur in slow-wave sleep (i.e., N3), which predominates the first third of the night. Nightmares typically occur during REM, with episodes more frequently observed in the second half of the sleep period. Recall is a critical differentiator; unlike nightmares, episodes of NREM disorders of arousal are not recalled. Behaviorally, in contrast to nightmares, NREM disorders of arousal are characterized by the dreamer appearing disoriented or partially asleep rather than fearful and oriented upon awakening.

Nightmares should also be distinguished from nighttime anxiety, nocturnal panic attacks, and sleep-related breathing disorders [most commonly obstructive sleep apnea (OSA); American Psychiatric Association, 2022, p. 460]. While these conditions may cause significant nocturnal distress, they lack vivid imagery recall. For example, OSA may be mistaken for nightmares when a child awakens suddenly gasping for breath because of an apneic event; in this case, fear arises from physiological distress, rather than dream content. Similarly, nocturnal panic attacks involve abrupt awakenings with intense distress and physiological arousal without dream recall (American Psychiatric Association, 2022, p. 460; American Academy of Sleep Medicine, 2014, p. 261). Nighttime anxiety in children is driven by environmental or situational fears (e.g., darkness, separation) and may lead to bedtime resistance or nighttime awakenings, whereas insomnia is characterized by fear of not sleeping, in contrast to nightmares, which are characterized by fear of sleep.

Nightmares have been categorized as either posttraumatic or idiopathic, a distinction derived primarily from research with adults (Youngren et al., 2024). Posttraumatic nightmare (PTN) onset occurs following a traumatic event; content can vary from replicative of the trauma to not bearing any similarity to a precipitant (American Psychiatric Association, 2022, p. 458). PTNs are associated with an inability to initiate the fear extinction process (McNamara et al., 2021), contributing to the development and maintenance of PTSD. In contrast, idiopathic nightmares have no identifiable precipitating cause and are theorized to initiate the fear extinction process; however, fear extinction is disrupted when the dreamer wakes prematurely (McNamara et al., 2021). In this way, idiopathic nightmares become negatively reinforced.

Evidence from pediatric samples is limited, but existing studies have explored whether nightmare etiology is associated with symptom severity and distress. Langston et al. (2010) found that youth with PTNs had more severe depression and PTSD symptoms compared to youth with idiopathic nightmares. Within a sample of youth with PTN, replicative nightmares were associated with more PTSD symptoms but not higher nightmare distress than were non-replicative nightmares (Gray and Cromer, 2018). Recently, Tett et al. (2025) extended this work to a broader sample of youth aged 6–17 years with either PTNs or idiopathic nightmares and found comparable nightmare distress and themes across groups. These findings raise the possibility that, in the developing brain, nightmares may represent a more unitary phenomenon, with trauma exposure and fear learning interacting along a continuum rather than constituting distinct etiological categories. This theoretical distinction is revisited in the next section on mechanisms of chronic nightmare maintenance.

## 3 Mechanisms underlying chronic nightmare maintenance

A theoretical breakthrough in behavioral sleep medicine was introduced by Spielman et al. (1987), who reconceptualized insomnia from a transient symptom to a learned, self-perpetuating disorder. Their model emphasized that behavioral and cognitive responses to poor sleep, such as compensatory sleep-interfering habits and worry about sleep, can maintain insomnia long after the initial precipitant has resolved. This work evolved into a 3P model to acknowledge predisposing, precipitating, and perpetuating factors in the development and maintenance of insomnia.

(Davis 2009, p. 63) extended Spielman's conceptual framework to explain PTN development and maintenance, highlighting the similar roles for predisposing vulnerabilities, precipitating or triggering events, and ongoing behavioral and cognitive perpetuating factors in the onset and maintenance of chronic PTN. (Davis 2009, p. 63) identified key perpetuating factors for nightmares including sleep deprivation and REM disruption, daytime distress and arousal, anticipatory anxiety, avoidance, and maladaptive sleep habits.

More recently, Sheaves et al. (2023) drew on network approaches to illustrate how nightmares may participate in reciprocal symptom-symptom interactions. The authors conceptualized nightmares as part of a broader system of feedback loops (e.g., nightmares increasing anxiety and hyperarousal, which in turn increase vulnerability to further nightmares) that may contribute to comorbidity. The authors suggested that treatment approaches may be more effective if they target central components within a network. By identifying and modifying feedback loops, interventions may disrupt the broader network that sustains nightmares. Nevertheless, there remains a need for a mechanism-focused framework that explains how symptom loops develop and clarifies which processes can be directly targeted to reduce chronic nightmares across etiologies.

Nightmare distress is an important contributor to nightmare maintenance. Belicki (1992) observed that negative daytime affect following a nightmare predicted poor psychological functioning. Levin and Nielsen (2007) proposed a model in which one's emotional load (i.e., cumulative emotional distress and ability to regulate it) becomes too great, causing a nightmare. Drawing on classical conditioning principles, they argued that adaptive dreaming ordinarily facilitates fear extinction: the feared memory (conditioned stimulus) is repeatedly reactivated in the absence of threat (unconditioned stimulus), leading to fear extinction. However, when affect load is high, this extinction process becomes disrupted, increasing the likelihood of a nightmare. The resulting nightmares then heighten next-day distress, especially in individuals with high dispositional stress sensitivity.

Gieselmann et al. (2020) applied a transactional model of stress and coping to understand nightmare distress. Primary appraisal involves evaluating whether a nightmare is threatening to one's well-being, followed by a secondary appraisal of one's coping potential. They found that individuals with higher nightmare distress engaged in more daytime coping efforts, suggesting more attempts to manage distress. Notably, greater distress was also reported among participants who believed they knew the cause of their nightmares, which the authors suggested may reflect heightened emotional salience of nightmares linked to identifiable stressors, such as traumatic experiences.

Rousseau and Belleville (2018) conducted a systematic review to explore the hypothesized mechanisms underlying psychological treatments for nightmares. They identified six mechanisms to explain treatment efficacy: increased sense of mastery, emotional processing and fear-structure modification, belief restructuring, restoration of sleep functions, reduction of physiological arousal, and prevention of avoidance behaviors. Among these, the enhancement of mastery, or patients gaining control over their nightmares, emerged as the most frequently cited mechanism. Notably, while mastery has been discussed in relation to treatment response, it has been less explicitly examined as a mechanism underlying the maintenance of nightmares. The remaining mechanisms fit with existing transactional models of stress (e.g., restoring sleep functions, reducing physiological arousal, preventing avoidance) and cognitive models, including 3P frameworks (e.g., fear-structure modification, belief restructuring).

Nightmares may arise from a variety of precipitating factors, including psychological stress (Nielsen and Levin, 2007), traumatic experiences (American Psychiatric Association, 2022, p. 458), pharmacologic influences, and psychiatric or medical comorbidities (such as sleep fragmentation from sleep related breathing disorders). However, the current model posits that while the precipitant might differ, once nightmares emerge, their persistence is driven by shared learning mechanisms. The etiological distinction is an interesting theoretical question because treating PTN may be akin to a trauma exposure; however, from a learning theory perspective, nightmare-related fear sustains avoidance-based cycles for both idiopathic and PTN. Furthermore, at least in youth, preliminary findings suggest that there are no functional differences between idiopathic nightmares and PTNs (Tett et al., 2025).

## 4 DARC-NESS: a nightmare mastery model

Building on prior theoretical approaches, the DARC-NESS model integrates elements of the 3P formulation (Davis, 2009, p. 63; Spielman et al., 1987), affect-load theory (Nielsen and Levin, 2007), transactional stress concepts (Gieselmann et al., 2020), and network perspectives (Sheaves et al., 2023) to explain how nightmares are maintained across etiologies. DARC represents the core maintaining processes, D: Dream (or nightmare) content, A: Appraisal, R: Regulation resources, C: Conditioned arousal, while NESS represents the central mechanism of Nightmare Efficacy and sleep-related maintaining factors including, S: Sleep hygiene and patterns, and S: Sleep quantity and quality. While nightmares themselves are understood as avoidance or escape from distressing dream content (Levin and Nielsen, 2007), nightmare maintenance is conceptualized as arising from dynamic interactions among cognitive, emotional, behavioral, and physiological processes that may be activated differently across individuals (Sheaves et al., 2023).

Consistent with Davis (2009), the model highlights perpetuating cognitive, behavioral, emotional, and physiological processes, but extends these ideas by allowing multiple entry points, rather than a single ordered sequence. This flexibility reflects a network-informed perspective in which processes interact non-linearly and vary across individuals (Sheaves et al., 2023). Drawing from Levin and Nielsen's (2007) conceptualization of affective overload, as well as work on coping resources in nightmare treatment (Gieselmann et al., 2020), affect overload is represented in the model as insufficient regulatory capacity relative to emotional demands. Finally, influenced by Rousseau and Belleville (2018), the model centers nightmare efficacy as the core mechanism underpinning the other components.

**Figure 1:**
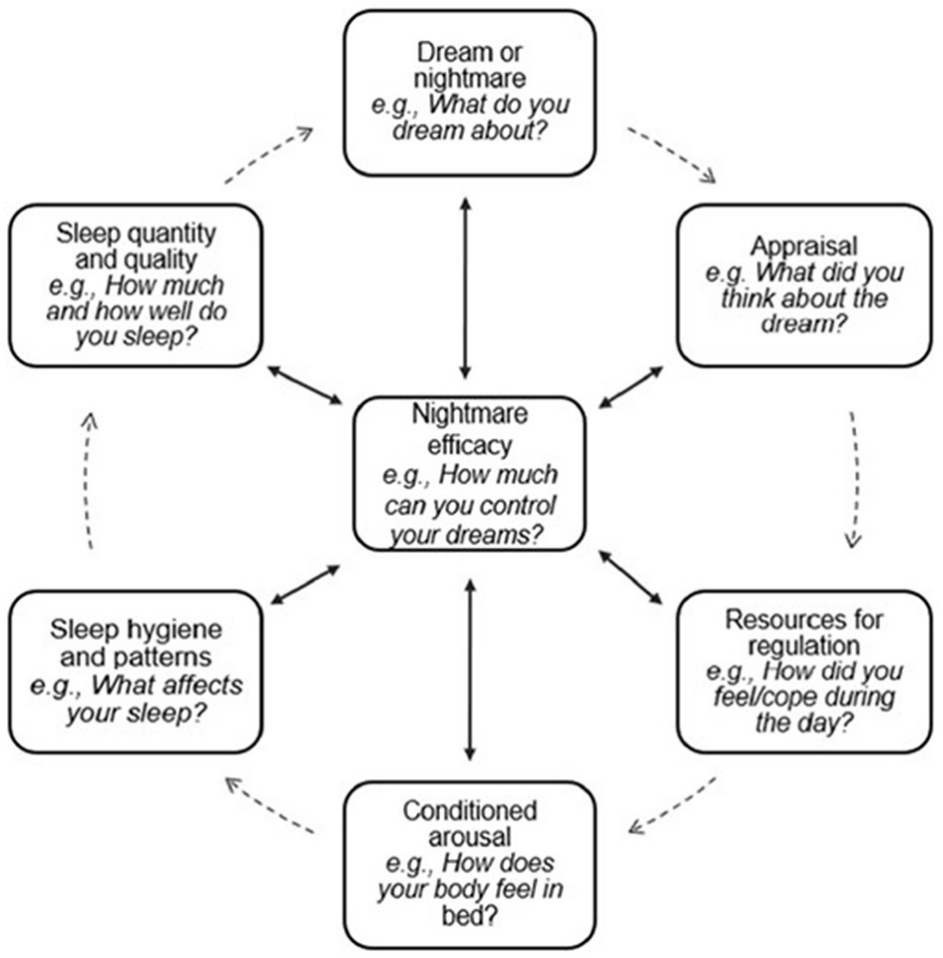
The DARC-NESS model.

The current model proposes that in clinical practice, helping children understand how nightmares are maintained is a critical therapeutic task. Nightmares are often experienced as confusing, uncontrollable, and frightening events (American Psychiatric Association, 2022), and children (and their caretakers) may not connect how daytime experiences, emotions, and sleep-related behaviors contribute to nightmare recurrence. Below, the DARC-NESS model is described both as a theoretical framework and a psychoeducational tool that can be used collaboratively with youth to explore how nightmares are maintained and how they can be changed; Figure 1 includes example clinician prompts that may be used to guide the process. The model provides a shared language for discussing nightmares, externalizes the nightmare pattern from the child, and allows clinicians and youth to identify which processes are most relevant for a given individual, rather than assuming a one-size-fits-all pathway. This interactive process guides collaborative treatment planning.

## 5 Nightmare maintenance pathways

The DARC-NESS Model in Figure 1 is depicted as a circular, multi-component system; clinically, this serves to help youth identify their personal cycle based on these pathways. The clinician should note, however, that the pathways are functionally non-linear, and not all youth may experience each component; this variability is represented with dashed lines. Youth may enter the cycle at any point and may bypass one or more components entirely, depending on the developmental level and individual experiences.

Each component of the DARC-NESS model represents a distinct process that can contribute to nightmare maintenance. Dream (nightmare) content maintains nightmares when they are emotionally salient or repetitive, increasing affect load, fear activation, and the likelihood of awakening to escape distress, as well as reactivation of fear networks across nights. Appraisals of nightmares as dangerous, uncontrollable, or indicative of personal vulnerability heighten perceived threat and anticipatory anxiety, promoting avoidance of sleep or premature awakening. Resources for regulation influence maintenance when a youth has limited capacity to manage emotions or physiological arousal, increasing vulnerability to affective overload and reducing tolerance of distressing dream content. Conditioned arousal reflects learned physiological reactivity to sleep-related cues which interferes with sleep initiation. The NESS components represent downstream processes that sustain nightmares by shaping both sleep processes and perceived agency, including nightmare efficacy, sleep hygiene and patterns, and sleep quality and quantity.

The components of the model represent common but not universal processes, and the core mechanism—nightmare efficacy—interacts dynamically with those processes. Nightmare efficacy reflects the individual's sense of agency, control, and belief in their capacity to cope with nightmares (Rousseau and Belleville, 2018). It functions as a central amplifying or buffering mechanism; low nightmare efficacy amplifies several interconnected maintaining processes, described below.

For clarity, treatment components are described below in the order of the DARC-NESS mnemonic rather than the order in which they are introduced in therapy.

### 5.1 Dream

Dream (or nightmare) content is at the orienting or “north” position of the model and is a common starting point for explaining one's nightmare cycle. The nightmare itself may function as a maintaining influence, particularly when dreams are vivid, repetitive, emotionally intense, or thematically linked to waking concerns, thereby increasing emotional salience and the likelihood of reactivation (Levin and Nielsen, 2007). Although nightmares involve exposure to distressing emotional material (Belicki, 1992; Levin and Nielsen, 2007), awakening during the emotional processing of sleep can serve as an escape. This interruption may contribute to the persistence of fear and distress associated with the nightmare (McNamara et al., 2021) and may reduce nightmare efficacy (Gieselmann et al., 2020).

### 5.2 Appraisal

Appraisal of the nightmare can contribute to maintenance (Gieselmann et al., 2020). Primary appraisal, in which a child perceives the nightmare as real and confuses dreaming with reality, risks heightening perceived threat, affect load, and hyperarousal (Gieselmann et al., 2020; Nielsen and Levin, 2007). The daytime worry or rumination creates a greater need for daytime coping resources (Gieselmann et al., 2020). Secondary appraisals may contribute to one's perceived inability to cope with or control nightmares (i.e., low nightmare efficacy; Gieselmann et al., 2020; Nielsen and Levin, 2007).

### 5.3 Resources for regulation

Although this component follows appraisal in the model, clinically our team has observed that poor regulation may occur in the absence of conscious appraisal, particularly in young children or those with less developed insight or self-awareness. Heightened daytime arousal can contribute to functional impairments (Sheaves et al., 2023). For example, children describe being in a bad mood at school, which can exacerbate interpersonal conflict or interfere with attention and classroom functioning. Young children may also experience dysregulation because of fragmented or insufficient sleep. Although appraisal and regulation resources often influence one another, the model does not assume a fixed sequence (Sheaves et al., 2023); in some youth, dysregulation may emerge in the absence of explicit cognitive appraisal, whereas in others, maladaptive appraisal may drive affective overload.

### 5.4 Conditioned arousal

Conditioned arousal results from a learned physiological response to sleep-related cues and represents a distinct maintaining process. Stimuli associated with the sleep environment, the bed in particular, may become conditioned cues if repeatedly paired with the distress of awakening from a nightmare. Over time, these cues may automatically elicit physiological arousal outside of conscious awareness (Gieselmann et al., 2020) even when the child attempts to sleep. In youth with frequent nightmares, cues such as being told, “It's time to go to sleep,” may be sufficient to trigger arousal, increasing sleep-onset difficulty and reinforcing the nightmare maintenance process.

### 5.5 Nightmare efficacy

Central to the model is the notion that awakening from a nightmare interrupts fear processing (Nielsen and Levin, 2007). Awakening acts as avoidance reinforcing both the occurrence of nightmares (Davis, 2009) and the belief that the individual cannot tolerate the feared content, thus lowering nightmare efficacy (Rousseau and Belleville, 2018).

### 5.6 Sleep hygiene and patterns

Limited regulation skills and conditioned arousal are often expressed through sleep hygiene and bedtime patterns (Spielman et al., 1987). Bedtime avoidance may manifest as safety behaviors (e.g., eating, seeking excessive reassurance), bedtime refusal, and inconsistent sleep routines. These behaviors may be accommodated by caregivers (Chevalier et al., 2021; Kendall et al., 2005), paralleling the perpetuating factors described in 3P models and PTN formulations and becoming negatively reinforced by short-term distress reduction (Craske et al., 2014; Davis, 2009). Poor sleep hygiene and behavioral avoidance may delay sleep onset or reduce total sleep time, thereby increasing physiological arousal and vulnerability to subsequent nightmares (Davis, 2009; Nielsen and Levin, 2007). Insufficient or fragmented sleep may increase REM pressure and the intensity of emotional dreaming, which may further elevate the likelihood of nightmares (Nielsen and Levin, 2007).

### 5.7 Sleep quantity and quality

Sleep characteristics contribute to nightmare maintenance by shaping the physiological conditions under which emotional dreaming occurs. Insufficient and fragmented sleep increase sleep pressure and alter REM sleep expression, which may intensify emotional dream content and increase the likelihood of nightmares (Nielsen and Levin, 2007). Recurrent awakenings further disrupt sleep continuity, shortening REM periods and increasing transitions between sleep and wake, conditions associated with heightened dream recall and emotional salience. Over time, reductions in sleep quantity and quality create a sleep architecture that favors repeated nightmare occurrence.

To translate the proposed nightmare-maintenance model into a clinically useful framework, we developed a multi-component nightmare mastery model that identifies the core maintaining processes and their corresponding intervention targets (Table 1).

**Table 1:** Nightmare-maintaining processes mapped to intervention targets.

**DARC-NESS component**	**Maintaining process**	**Therapeutic target (mastery pathway)**
Dream/nightmare content	Vivid, repetitive, emotionally intense dreams that trigger awakening and avoidance	Nightmare rescripting, exposure to dream content, tolerating affect
Appraisal	Interpreting nightmares as real, dangerous, or uncontrollable	Psychoeducation, neutral/accurate appraisal, cognitive reframing
Resources for regulation	Limited emotion regulation skills; high daytime affect load	Regulation skill-building (relaxation, mindfulness, coping tools)
Conditioned arousal	Bed and bedtime cues associated with fear and physiological arousal	Deconditioning, relaxation in bed, stimulus control
Nightmare efficacy (central)	Low perceived control or coping capacity	Mastery experiences, skills practice, agency-building
Sleep hygiene and Patterns	Bedtime avoidance, inconsistent routines, safety behaviors	Consistent routines, sleep hygiene, reduced avoidance
Sleep quantity and quality	Fragmented or insufficient sleep increasing vulnerability to nightmares	Sleep consolidation, improved duration and continuity

#### 5.7.1 A nightmare mastery model

Within the DARC-NESS framework, nightmare mastery involves disrupting the maintaining processes described above and transforming them into pathways that support adaptive sleep and emotional processing. Consistent with the network-informed nature of the model, mastery may be achieved through multiple entry points rather than a fixed sequence of steps.

Clinically, the model can first be used to describe nightmare-maintenance processes to youth and to elicit their own working model of how their nightmares are maintained. Following this formulation, the clinician and youth jointly identify an entry point for intervention. This collaborative process works well with a dynamic model and fosters engagement, buy-in, and a growing sense of agency. Across pathways, therapeutic change is unified by increases in nightmare efficacy (i.e., the youth's belief in their capacity to influence, tolerate, and ultimately change their nightmare experience). The multi-component nightmare mastery model pairs each maintaining factor with its therapeutic counterpart, with efficacy serving as a common mechanism across components.

### 5.8 Intervening with the dream (nightmare) pathway

Dream/nightmare content is typically not addressed first in the mastery-based formulation. Unlike imagery rehearsal/rescripting therapies which directly target the nightmare content early in treatment (Krakow and Zadra, 2006; Gieselmann et al., 2019), the DARC-NESS model prioritizes building efficacy through experimentation with the other components of the model. Once efficacy has been strengthened, nightmare content is addressed by collaboratively selecting the most distressing nightmare. Drawing on skills and insights developed in earlier sessions, the clinician supports the youth in detailed exposure to the dream content, and subsequently, in rescripting the nightmare.

Consistent with Davis (2009), Fernandez et al. (2013), and Cromer et al. (2024), the nightmare is described in detail in the present tense, incorporating sensory experiences across modalities. After identifying a central nightmare theme, the nightmare is rescripted in a manner that retains similarity to the original while modifying elements so that the youth would want to dream the revised version; functionally, the clinician ensures that the upsetting theme has been directly addressed (Cromer et al., 2024; Davis, 2009).

With any exposure, the clinician should remain supportive and encouraging (Craske et al., 2014; Kendall et al., 2005). In our work, there has been considerable variability in how visibly upset (or not) a youth is during exposure. When a youth becomes tearful or distressed, the clinician may respond supportively by validating the fear (e.g., acknowledging that the nightmare is frightening) and, using their clinical judgment (Kendall et al., 2005), reinforcing the youth's bravery and ability to tolerate the distress. Similar to conducting anxiety exposures (Kendall et al., 2005), clinicians may also normalize the distress by acknowledging the aversive nature of the content while emphasizing the value of externalizing the nightmare through drawing or writing, effectively getting it “out of their heads, and onto paper.”

### 5.9 Modifying the appraisal pathway

Negative appraisal is countered with psychoeducation about nightmares, normalization, and neutral appraisal. Education about the nightmare cycle and the multiple ways it can be disrupted can be empowering, even for young children, by increasing understanding and perceived coping capacity (Kendall and Hedtke, 2006). Caregivers and youth commonly report that this empowers youth as they learn that they can take active steps to manage their nightmares (e.g., a mother of a 9-year-old noted that her child developed a “more positive mindset, because she learned she could do something about it”). Appraisal can be addressed as early as informed consent to treatment and is reinforced whenever psychoeducation about other components of the model is provided. In keeping with the model, clinicians explicitly link each skill or tool to increases in nightmare efficacy, explaining that it can help disrupt the cycle. Consistent with cognitive behavioral therapy (CBT) protocols, the clinician can encourage an open mind, curiosity, and experimentation about the effectiveness of skills, which reduces the risk of catastrophic interpretations if a particular strategy is not effective (Craske et al., 2014). Psychoeducation also provides a context for intervening at both primary and secondary levels of appraisal (Gieselmann et al., 2020).

### 5.10 Improving resources for regulation pathway

In our work, teaching youth multiple skills for regulation—building a regulation resource toolkit—has been effective for nightmare treatment. Across sessions, youth are introduced to a range of mindfulness and relaxation strategies, including belly breathing, box breathing, and developmentally appropriate progressive muscle relaxation (PMR; e.g., scripts that invite children to tighten their hand as if they were squeezing a lemon or tighten their tummy like a dog is about to jump on it), which are engaging and relatable. These skills often support improvements in bedtime routines by reducing physiologic arousal, and youth frequently report using them during the day to improve emotion regulation, reinforcing a sense of efficacy over mood states. In exit interviews, caregivers have often commented that they appreciated learning multiple tools, noting that “no one thing works all the time, but we always could find something in our toolbox to work each time!” (mother of a 12-year-old boy).

Cognitive regulation skills are also helpful for many youth, particularly when nightmares are associated with daytime worries (e.g., exams or peer difficulties). In these cases, interventions for anxiety (Kendall and Hedtke, 2006), such as a worry box, scheduling worry time during the day, and other cognitive strategies for managing anxiety, can reduce affect load. Mindfulness and breathing techniques support this component of mastery within the model. To support learning, regulation skills are framed as experiments (Craske et al., 2014) to observe whether they influence nightmare frequency or distress. By partnering with youth as “sleep detectives” who collect observations and report back, clinicians foster a growing ability to connect emotional regulation and sleep.

### 5.11 Addressing the conditioned arousal pathway

Conditioned arousal can be targeted both implicitly and explicitly. Borrowing from CBT for insomnia, the clinician may introduce deconditioning behavioral strategies such as a flexible “15-min then reset” approach, avoiding clock watching, or using words like “rule” which can provoke anxiety and rigidity (Harvey, 2002). Youth are encouraged to get out of bed briefly if unable to fall asleep, engage in a calming activity (e.g., writing a worry and placing it into the worry box before setting it aside), and then return to bed once arousal has decreased (Perlis et al., 2005). Using PMR or belly breathing in bed supports alternative learning (i.e., associating the bed with relaxation; Perlis et al., 2005). In our clinic, we have used other creative interventions, such as giving the youth an opportunity to use fabric markers to draw or write positive imagery or cues on a pillowcase. This activity is popular across age groups; as one 15-year-old boy stated, “it made me calmer because I was looking at things I actually know, and it kept me grounded.”

Bedtime avoidance is addressed by replacing unhelpful sleep patterns with sleep hygiene and healthy sleep routines (Davis, 2009; Spielman et al., 1987). Delayed sleep onset is reduced through relaxation and worry reduction (Perlis et al., 2005). Sleep routines are often a natural early entry point with youth and are associated with improved sleep onset (Mindell and Williamson, 2018). When youth report not having a bedtime routine, clinicians can explore what typically occurs before bed. In our experience, most youth do have bedtime routines, but these may consist of arguments, video games, or other deeply ingrained habits that interfere with sleep. Bringing these patterns to light allows them to be gently modified. When routines are paired with arousal regulation skills, youth often experience greater success in winding down, shifting the focus toward their own efficacy instead of, as one parent put it, “a fight with me to get him to bed.”

### 5.12 Developing nightmare efficacy

Throughout treatment, youth develop a growing sense of agency, the belief that their actions can influence their sleep and nightmares. The behavioral expression of efficacy is mastery, reflected in youths' confidence using strategies to manage nightmares. When youth experience success with a tool, it is essential that the clinician reinforce the learning as it relates to improving not only sleep but specifically nightmares. Mastery is often reflected in exit interviews; caregivers frequently describe their youth as “empowered.”

Clinicians cultivate agency by framing each new skill as an experiment in which youth try a strategy, observe the outcome, and evaluate its usefulness. Socratic questioning and a stance of curiosity help prevent catastrophic thinking (Craske et al., 2014). Logs of nightmare occurrence and distress level should be completed daily so that clinician and client can collaboratively explore what occurred during the day (e.g., stressors), how the youth responded (e.g., coping strategies), and ultimate outcome (i.e., nightmare presence or absence and distress level). This process promotes connections between actions and outcomes, reinforcing mastery.

### 5.13 Improving sleep hygiene and patterns

From a mastery-oriented perspective, sleep hygiene and bedtime patterns represent modifiable entry points for strengthening nightmare efficacy. Treatment focuses on collaboratively replacing avoidance-based behaviors with consistent, developmentally appropriate routines that promote sleep onset and continuity, while gradually reducing safety behaviors and caregiver accommodation (Chevalier et al., 2021; Spielman et al., 1987). Bedtime passes (Moore et al., 2007) may support caregiver limit setting. We have found that caregivers are often surprised when their youth express interest in implementing a bedtime pass program. Clinicians should remain attentive to different cultural norms and values when discussing co-sleeping, as independent sleep should not be an assumed goal.

As youth and caregivers experience success implementing changes, bedtime becomes more predictable and tolerable, reinforcing a growing sense of mastery. While the immediate focus may be general improvements in sleep, clinicians should be generalizing the planning and outcome monitoring to nightmares.

Improvements in sleep hygiene and reductions in bedtime avoidance may lower physiological arousal at sleep onset, reduce sleep fragmentation, and mitigate REM pressure, thereby decreasing the intensity of emotional dreaming and vulnerability to subsequent nightmares (Nielsen and Levin, 2007). As conditioned arousal at bedtime diminishes, attention to feared stimuli is reduced and secondary appraisals of helplessness or inability to cope may soften (Gieselmann et al., 2020; Spielman et al., 1987). Together, these changes support a downward shift in affect load at sleep onset, allowing sleep quantity and quality to improve and reinforcing nightmare efficacy over time.

### 5.14 Improving sleep quantity and quality

Within the mastery-based model, sleep quantity and quality represent both an outcome of treatment and a modifiable pathway for mastery. Because insufficient or fragmented sleep can amplify affect load and increase vulnerability to nightmares (Nielsen and Levin, 2007), treatment aims to strengthen sleep continuity and adequate sleep opportunity by consolidating sleep schedules, supporting consistent wake times, and reducing nocturnal disruptions. Clinicians explicitly link these changes to nightmare mastery by helping youth track sleep duration and night awakenings alongside nightmare frequency and distress, reinforcing perceived control when improvements occur.

#### 5.14.1 Case illustration demonstrating the DARC-NESS model

The following case illustrates how these interconnected components present and apply clinically. The youth, referred to by the pseudonym “Alex,” was a 15-year-old White transgender boy using he/him pronouns. At intake, Alex and his mom endorsed a history of multiple interpersonal traumas, including physical abuse, sexual assault by a peer in elementary school, exposure to a parent's severe mental illness, and witnessing domestic violence. Alex was hospitalized several months prior to baseline due to a suicide attempt and subsequently received therapy and psychiatric care for an unspecified mood disorder with psychotic features. Other diagnoses reported by the family included gender dysphoria, specific learning disorder with impairment in reading (dyslexia), sensory processing disorder, and a rule-out of autism spectrum disorder. Ongoing challenges for this family included economic hardship and moving midway through the therapy. Incentives of $10 for the parent and small gifts (value of $5) were sent to the youth for each week of participation. This treatment occurred through the course of a larger research study which had university Institutional Review Board approval; the family consented to anonymized information being shared for publication.

**Figure 2:**
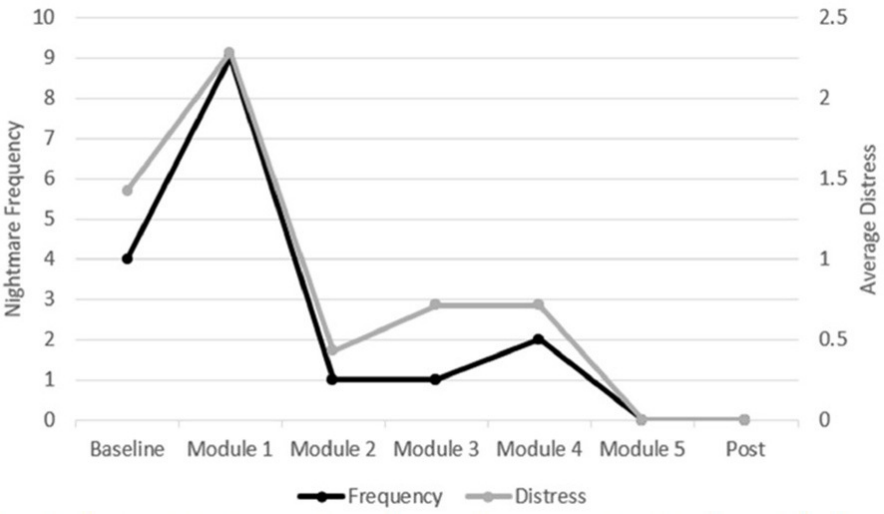
Alex's nightmare frequency and average distress change across treatment. Each timepoint relates to the nightmare diaries completed in the week leading up to the module.

At intake, Alex was taking escitalopram 15 mg daily and quetiapine 125 mg at bedtime, and medication dosages were stable for several months prior. He reported using no other over-the-counter medications. During intake, Alex disclosed having suicidal thoughts while waiting to fall asleep at night. He denied having a current suicide plan or intent. A Structured Clinical Interview for DSM-5-TR Sleep Disorders (SCISD)–Kid (Rischard et al., 2024) was conducted. He screened positive for insomnia and nightmare disorder. Multiple weekly nightmares had occurred for at least 7 years; sleep diaries revealed four nightmares across three nights with an average daily distress of 1.57 on a 0–5 scale (5 indicating extremely upsetting). See Figure 2 for values across treatment.

Alex and his mom described a close, supportive relationship. Nightmare treatment was concurrent with ongoing weekly mental health treatment through a community provider. Alex's mother checked in at the beginning and end of each session to offer support and encouragement. All sessions were conducted over a secure hospital-owned Zoom account, with Alex and his mother in their home.

The first session began with psychoeducation about cognitive-behavioral therapy and a review of Alex's nightmare cycle to introduce how nightmares can create a spiraling effect in daily life. The clinician drew a spiral and asked Alex to describe how he felt upon waking from a nightmare and what typically happened next. Alex's nightmare spiral revealed that when he woke up from a nightmare and was able to fall back asleep, he often continued the same nightmare. He described associated physical discomfort, stating, “Sometimes the nightmares cause physical pain, and it makes my body sore.” After a nightmare, Alex reported difficulty getting up for school and feeling tired all day. By evening, he noticed blurry vision and poor concentration, which ultimately made reading harder. He also described heightened frustration and more arguments with family members. Finally, Alex noted that it was harder to fall asleep if he had had a nightmare the previous night.

The clinician used this spiral to motivate Alex for change by highlighting how treating nightmares could improve other valued areas of his life. This motivation can later be revisited using a motivational interviewing stance if resistance emerges around reducing avoidance, improving sleep habits, or engaging in exposure and imagery rehearsal. Introducing an interconnected model of thoughts, feelings, and behaviors helped established expectations for collaborative, trial-and-error learning and supported efficacy and curiosity. The clinician then invited Alex to identify where he would like to begin in the cycle.

To further support motivation, the clinician engaged Alex in goal setting by asking him to imagine what life might be like without nightmares. Alex shared that he would feel less sleepy at school and drew a simple happy-face emoji.

Given Alex's difficulties, they first focused on building resources for regulation. In this initial session, Alex learned a brief belly-breathing exercise, which was practiced together. Then, they identified daytime situations in which belly breathing might be helpful, including moments of peer conflict or when others were being unkind.

The clinician also introduced the importance of a consistent bedtime routine to prepare for sleep, directly targeting the sleep hygiene and patterns component of the model. Alex described his existing routine as watching “satisfying” videos (e.g., pimple-popping), eating a snack, changing into pajamas, brushing his teeth, reading, and curling into a ball with a weighted blanket. However, he routinely slept with a pet and voiced a belief that it was the only way he could fall asleep.

Alex reported waking at 7:00 a.m. for school and identified a desired bedtime of 9:30–10:00 p.m., leading to a plan to begin a structured bedtime routine at 9:00 p.m. Together, Alex and the clinician developed a revised routine that included turning off his phone at 9:00 p.m., watching a relaxing show, eating a snack, brushing his teeth, getting his blanket, belly breathing, and going to bed with a cat. While waiting to fall asleep, Alex planned to listen to calming music or nature sounds (e.g., rain). He committed to completing the bedtime routine nightly and tracking his sleep and nightmares over the following week.

At the second session, Alex reported following his bedtime routine consistently. He said that belly breathing calmed him and he fell asleep faster. Overall, his sleep was reportedly more restful, and he had only one nightmare. Alex attributed these improvements to reduced stressors and to consistent use of belly breathing, suggesting early disruption of the nightmare maintenance cycle through enhanced regulation resources. His report that he had less stress may also reflect an improvement in appraisal.

The second session included psychoeducation emphasizing the importance of relaxation before bed as a means of preparing the mind and body for sleep, targeting the sleep hygiene and patterns component of the model. Avoidance was explained as a nightmare maintenance mechanism and “avoiding avoidance”—that is, preparing intentionally for sleep—was introduced to support sleep health. This framing resonated with Alex, who recognized his pattern of delaying bedtime due to fear of nightmares. He described staying up late reading past his ideal bedtime, keeping the television on for distraction, and sleeping with a pet or parent as safety behaviors. The clinician reframed these behaviors as unintentionally increasing vulnerability to nightmares; in response, they collaboratively refined his bedtime routine. Alex planned to replace screen time with relaxing activities such as painting or spending time with his cats. He also decided to stop sleeping with his cat to practice self-soothing rather than relying on a safety behavior. This distinction was discussed explicitly, noting that while sleeping with a pet may be adaptive for some, in Alex's case not sleeping with a pet could support an internal sense of control.

To address nightmare-related rumination, the clinician introduced a worry box. Within the model, the worry box targets multiple components by supporting daytime regulation, reducing pre-sleep worry linked to conditioned arousal, and limiting worry in bed that can interfere with sleep hygiene. A small cardboard box and markers were mailed to Alex's home so he could decorate it during session. Alex drew animals and colorful flowers on the box and prepared slips of paper for recording worries. His worries were about having another mental health crisis, rehospitalization, and concerns about his pets' health.

Alex and the clinician scheduled worry time during the morning before school, and Alex planned to keep the worry box on the living-room windowsill and set a morning reminder. He also planned to incorporate yoga into his daytime routine. These changes added additional regulation resources as Alex actively shaped his coping strategies (Kendall and Hedtke, 2006).

In the third session, Alex was pleased that he again only had one nightmare that week, despite a severe stressor (i.e., a family argument). Alex explained that he “put [his] worries away” in the worry box; nonetheless, the one nightmare was the night of the argument. He viewed it as meaningful progress that he did not experience additional nights with nightmares. Alex continued to use the belly breathing daily, stating that, “I've become a lot calmer, and it's actually started to help me a lot when I wake up.” These changes suggest continued improvement in regulation and sleep hygiene, as well as possible reductions in conditioned arousal, though the latter was not explicitly reported. Moreover, Alex appeared to be developing a sense of control over his nightmares through the effective use of these skills.

Two additional skills were taught in the subsequent session. To expand Alex's relaxation repertoire, PMR was introduced to help with daytime distress and conditioned arousal before bedtime. They practiced PMR together for about 8 min. Although Alex was engaged, lying on his bed with his eyes closed and following the script closely, he had a leg cramp. They paused until it passed and modified the script to be upper body only. Alex reported slightly increased muscle tension afterward and noted that the most helpful aspect of PMR was deep breathing. The clinician used this moment to reinforce the trial-and-error nature of skill-building, and an attitude of openness. Alex agreed to try PMR again, and then they would evaluate its effectiveness the following week.

To further reduce conditioned arousal at bedtime, the clinician introduced the idea that the brain can be cued toward more pleasant dream content. Alex and the clinician brainstormed “movies” he could create with his imagination to play in his mind while he tried to sleep. His ideas focused on cats and aquatic animals, all which reportedly made him feel safe; he also wanted to cue memories of being in nature (e.g., walking in a forest), and outdoor adventures such as hunting, fishing, and rock climbing. Then, Alex used fabric markers to draw on his pillowcase; it had a mountain scene with trees, birds, the sun, and several cats. Alex said, “We're gonna use [the pillowcase] to think about good things to dream about.”

At the start of the fourth session, Alex was not enthused. He was sitting on the couch next to his mom, petting his cat, and appeared tired. He had had two nightmares that week; although this was more than the previous week, he said they were less distressing. He said that his routine had been off because of a 3-day weekend, plus he had been sick and missed school. The clinician was empathic, normalized fluctuations in energy and motivation, and they agreed to reschedule. However, before ending the appointment, they reviewed his therapy homework.

He said he enjoyed the belly breathing and worry box but disliked the PMR due to “it making [his] body sore.” He said, “[PMR] didn't feel good.” The clinician praised Alex for his willingness to try it and framed this as part of the mastery process, emphasizing that learning what does not work is also valuable. He had not used the pillowcase because the artwork was unfinished, and his mother had put it in a closet out of sight. The clinician and Alex problem-solved ways to reintroduce the pillowcase and reviewed his plan for belly breathing and his bedtime routine.

The following week, Alex presented as more like his usual self. He reported one nightmare that week, with a distress rating of 5/5. He attributed the nightmare to increased anxiety after learning that his family planned to move and that some of their cats would need to be rehomed. His mother added that Alex had a dream in which one of the cats got hit by a car. Despite this stressor, Alex reported using skills from treatment, including the worry box and pillowcase, and both he and his mother believed these tools helped prevent escalation of nightmares. Alex proudly shared his now-completed, vibrant pillowcase, further illustrating the value of addressing multiple components of the model to strengthen self-efficacy.

During this fifth session, the clinician planned for Alex to directly address the nightmare. A sense of nightmare mastery is explicitly cultivated as the clinician explains to the youth that by facing the fear and talking about the nightmare out loud, it helps the brain know that it can tolerate the information and stay asleep for fear processing to occur. In other words, the mechanism of emotion processing and fear extinction during sleep is shared in therapy, as is the theoretical mechanism that maintains nightmare–negative reinforcement and escape.

Efficacy is cultivated by first invoking a mastery-based memory of overcoming a fear. Alex said he used to be afraid of the dark, imagining scary shadowy figures and things lurking in the dark, but he said those fears are gone. He voiced a belief that fears are in the mind, and if he could face his fears, he could sleep better. The clinician used this as a jumping off point to explain that together they would face his scariest nightmare together.

To start this process, the clinician gave an example of someone else's nightmare, first showing one that was brief with very little detail and in the past tense and then another one that was the same storyline but with much more detail and in the present tense. Alex read both and discussed the differences with the clinician as a learning moment that we want to activate as much of the memory of the feared nightmare as possible through details, using all five senses, and present tense. Alex stayed engaged in the discussion but appeared to look down and doodle during the exposure discussion, which may have represented avoidance. He was able to quickly identify his scariest nightmare which he named “The Eyes” because of its recurrent content. He rated the nightmare distress severity 8/10, at which time, he called his cat onto his lap, apparently for comfort.

As Alex settled, he started to share his nightmare story; at this point, the clinician was sharing her Zoom screen so Alex could see her typing his narrative. His nightmare began by him “waking in a pitch-black room that is completely void with nothing inside.” The nightmare exposure continued: “I feel fear growing inside. I search for a light, but can't find one, and I am even more afraid. I hear laughter in the distance but can't see the source; now I'm even more afraid. I can smell metallic burning human flesh. I find a light switch near a strange groove that is slightly wet. When I turn it on, I am surrounded by eyes all around me, peering at me. This, along with the smell of burning flesh, is intolerable. I realize I am all alone and standing in a pile of disgusting human flesh. It is throbbing beneath me. I try to escape, but my foot is stuck inside a large eye. I panic. The other peering eyes look dead and start to move toward me. I am shocked when I hear a voice overhead; it asks why I am in ‘the room of the disturbed.' The voice gets more demanding, repeating the question louder and louder. I can't speak and can't escape. The walls of the room start to move toward me, and chains come out of the floor and wrap around my throat. They drag me to an open door; I am not in control of my body and am overcome with fear. I am losing my mind! I struggle against the chains, and that's when I wake up.”

The clinician, while balancing the directness and compassion necessary for an exposure (Craske et al., 2014), gently tells Alex that she would read it through to see if she got the details correct. This is a unique aspect of exposure in CBT for nightmares in children (Cromer et al., 2024); it allows for an exposure at a distance because attention is focused on semantics rather than getting caught up in the story. She reads through, Alex has no corrections, and when asked how distressed he feels, he reports 5/10. Then, she asks Alex to read the story himself, aloud, and, following that, he reports a distress of 3/10. Exposures facilitate fear processing and provide a mastery experience to promote efficacy; the youth learns that the fear is survivable (Craske et al., 2014). By extension, as the clinician relates this to nightmare mastery, and theoretically, the newfound tolerance of the nightmare reduces affect load to allow fear processing during REM (Nielsen and Levin, 2007).

After the nightmare exposure, the clinician explained the next step was to identify the theme or main idea within the nightmare. This will be central as the youth changes the story into a version they would like to experience and should be explicitly addressed in the revised narrative (Cromer et al., 2024). This approach is grounded in trauma processing theory (Resick and Schnicke, 1992), and was adapted by Davis (2009) for PTN. Although the current treatment is also used for idiopathic nightmares, the process of evaluating the nightmare theme provides an opportunity to promote workability (appraisal in DARC-NESS), approach (resources for regulation), and efficacy when engaging with the nightmare.

Alex was presented with the different themes: safety, trust, power/control, esteem, intimacy (Davis, 2009; Fernandez et al., 2013) and escape (Cromer et al., 2024; Gray and Cromer, 2018). He said the two relevant themes were powerlessness and escape. Alex kept the first part of the narrative the same. He still described it as feeling fear, initially being unable to find the light switch, smelling flesh, and his foot being “stuck in a large eye.” Then, he started to make the change. He did not describe feeling panicked and when he heard the voice overhead asking why he was in “the room of the disturbed,” he found his voice. The narrative was: “I tell the man that I am not supposed to be here. The man responds, “Of course you are not, that is why I am asking you why you are here.” I tell him that I am having another dream and that I didn't mean to disturb the eyes. The man explains loudly, but in a friendly manner, “Well then why didn't you say so?” He then quietly opens a door and I can see him standing there. The man's body is soft and gentle. He has soft features, black hair, and emerald-green eyes that glow. His eyes are normal, unlike the ones on the wall. I feel a type of peace, while the man extends his hand and gestures for me to take it. He slowly guides me back out of the room with the eyes. I feel calm and in control.”

After rescripting, Alex's distress was 1/10. His body appeared relaxed and he smiled. He was playful when his mother rejoined the session and proudly shared the rescripted dream with her. The clinician encouraged Alex to read the new dream before bed each night, and they made plans for Alex to rescript any nightmares that might occur in the coming week.

To further augment Alex's resources for regulation, the clinician, together with Alex, practiced a slow breathing technique, in which they breathed in through the nose for 3 s and slowly out through the mouth for 6 s. They did this for three cycles, and Alex smiled broadly, appearing relaxed, despite having nasal congestion that day. He planned to use the new breathing technique during the day and before reading the new dream before bed.

At the sixth and final appointment, Alex reported that he was happy with the rescripted narrative and reported no nightmares that week. Of note, he had not read the new narrative before bed. Because this was the final planned session for nightmare treatment, the clinician did several things for maintenance planning and to bolster efficacy. First, she showed Alex and his mom a graph of Alex's nightmare frequency and distress (taken from sleep logs) from intake to the current week. The clinician and Alex explicitly reviewed which skills Alex had used to create the effects on the graph. Next, they reviewed Alex's nightmare maintenance spiral from the first session and discussed how he broke the cycle. Alex said, “I was able to strengthen my mind to be able to grow and be more aware of the nightmares rather than trying to block them out. You guys helped me see what exactly happens, why exactly it happens. Being able to learn about why it happens allows you to heal why it happens. I am in power now.” This statement reflects a growing sense of nightmare efficacy for Alex; he was now in control and felt powerful. He also referred to himself as being a “pro” regarding his therapy skills. He referred to “flipping the nightmare spiral,” indicating his mastering over breaking the maintenance cycle.

Alex reported that due to not having nightmares that week, his sleep was uninterrupted and he awoke refreshed each day. He commented that “waking up was easier; I was able to pay attention better at school; and I was less irritable because it was easier to fall asleep at night.” Compared to when he started, he reported that he felt “more at peace which allowed me to perform better.”

Next, the clinician asked Alex to identify which skills he planned to use in the future if nightmares occurred again. This intentionally prevents catastrophizing if there is a recurrence (appraisal) and promotes mastery. He said the most helpful parts of the therapy were the deep breathing techniques that he felt everyone should have in their toolkit. He also enjoyed the pillowcase because it directs his mind to positive thoughts before bed, and that he learned that “without self-control and knowing what's wrong with your body, you won't really be able to function properly.” He then drew a toolkit with his favorite activities, including worry box, deep breathing, and the pillowcase. Additionally, the clinician encouraged him to practice nightmare rescripting with future nightmares.

Finally, the session ended with a guided imagery technique that could be used for daytime regulation or relaxation before bed, helping with conditioned arousal. The clinician played a recording that involved walking along a riverbed, sitting on the riverbank, and mindfully placing any thoughts that popped up, on a leaf and watching it float down the river. Alex liked this guided imagery activity and said he felt very calm afterwards. He planned to use it to manage distress after conflict at school.

Following study completion, Alex and his mother participated in a follow-up phone call to provide feedback on their experience. Alex reported that reductions in nightmares allowed him to feel better at school and in daily activities, and that he no longer had suicidal thoughts. His mom observed increased positive affect (i.e., he was smiling more), reduced anxiety, improved sleeping, greater self-confidence, and less bedtime avoidance. Alex agreed and attributed these changes to no longer worrying about nightmares. He said, “I used to have a lot of nightmares which usually impacted my whole day and made me stressed to go to sleep again. Now that's not an issue.” Alex's mother also said that he “is opening up and talking about what bothers him more. And I don't think that worrying thoughts are sitting in his brain.”

Alex shared that his favorite parts of therapy were the pillowcase, deep breathing, and worry box. He said, “Every time I see my pillowcase, I'm like, “Oh my god, I could go on an adventure someday!”, and then I end up dreaming about adventures. So, it did what it was supposed to.” He went on to say, “I think that one of the things that helps the most is feeling in control of your nightmares. If you think, “No, I'm not going to have a nightmare tonight and that I'm just gonna sleep,” you're just gonna sleep. Your mind listens. Yippee.”

## 6 Clinical applications

This case was selected to demonstrate the treatment model and its clinical application, given the adolescent's engagement in therapy and the breadth of nightmare content addressed through rescripting. In addition, the case illustrates common clinical challenges, including variable homework adherence (e.g., pillow case and reading the new dream narrative), the need to discontinue or modify ineffective strategies (i.e., PMR), and the adaptability of the intervention in the context of significant life changes, such as relocation. The therapy modules represent a range of therapeutic activities that can be implemented flexibly and non-linearly in response to individual needs and circumstances.

A thoughtfully constructed case formulation enables clinicians to design a treatment plan that targets the theorized mechanisms of change (Eells, 2025). The multi-component model of nightmare maintenance is a valuable framework for guiding case formulation of nightmares and thus treatment of nightmare disorder. A detailed sleep and nightmare assessment is critical for identifying the relevant components in a child's case formulation. A clinical interview with both the child and caregiver can reveal which components are most relevant to the child's nightmare maintenance. It is important for the clinician to get a detailed picture of evening routines, sleeping environment, caregiver responses to the child's nighttime behaviors, as well as typical sleep schedule parameters including time in bed, sleep onset latency, wake after sleep onset, wake time, and daytime sleep (Mindell and Owens, 2015). Sleep and nightmare diaries are invaluable for supporting assessment and monitoring change. Additionally, as demonstrated with Alex, that data can then serve as a tool for reinforcing a child's self-efficacy by connecting cognitive-behavioral changes to observed changes in outcome. Standardized assessments may also be useful depending on the clinical setting; for example, broad screening tools such as the Structured Clinical Interview of Sleep Disorder in Children (Rischard et al., 2024), can help identify comorbid medical sleep disorders (e.g., OSA) that require further evaluation.

Once an initial formulation is developed, it is important to share this working understanding with the child in a developmentally appropriate way. In our work treating nightmare disorder, we have found that a visual (like the example in Figure 1) and describing a “vicious cycle of nightmares” is helpful. During this discussion, the clinician presents the cycle and the components most relevant to the child, emphasizing that the cycle can be broken at multiple points with the use of tools or coping skills learned in treatment. This discussion helps cultivate hope and motivation for change by underscoring choice in selecting from multiple pathways to improvement.

With a shared and discussed case conceptualization, clinicians can then identify relevant interventions based on the theorized components relevant for the child. Selected interventions are aimed at addressing the underlying mechanism of the relevant component. For example, when addressing conditioned arousal, the relevant intervention technique can include introducing the child and caregiver to stimulus control principles (Spielman et al., 1987; Perlis et al., 2005). These strategies can be implemented developmentally; for instance, psychoeducation about conditioning paired with creating a pillowcase with positive reminders can serve as a helpful cue for children that the bed is a place for sleep and relaxation. Table 1 summarizes model components alongside corresponding intervention targets and is intended as a flexible, non-exhaustive guide highlighting evidence-based strategies for treating nightmare disorder.

After identifying relevant components and corresponding interventions, the sequence of modular components may be tailored to the child's presentation. This modular approach is flexible: interventions may be delivered sequentially, concurrently, or iteratively depending on factors such as nightmare severity, treatment goals, readiness, and comorbid conditions. For example, in an integrated behavioral health setting such as a pediatric sleep clinic, it may be appropriate to begin by addressing hyperarousal with relaxation techniques or implementing a bedtime routine before engaging with nightmare exposure. Conversely, a child being seen in a more traditional behavioral health setting that may already have an established therapeutic rapport may be able to engage in nightmare exposure earlier.

Clinicians should view nightmare case conceptualization as a hypothesis, meaning it is testable and modifiable, modeling an empirical stance of curiosity and openness. Progress monitoring is essential for examining treatment response as well as for building self-efficacy by linking behavioral changes to improvements in sleep and nightmares (Craske et al., 2014; Rousseau and Belleville, 2018). If core components do not reduce nightmare frequency or distress, it is important to revisit the initial formulation and consider other factors, such as co-occurring medical sleep disorders.

Not all youth experience complete remission of nightmares; however, most show meaningful improvements, with gains continuing beyond treatment completion. If nightmare frequency or distress does not improve after addressing the youth's identified maintenance factors and completing two rescripting sessions, a referral for medication treatment of the nightmares may be warranted. Our team has found that nightmare rescripting has not caused iatrogenic effects. That said, while youth may need rewards and motivations to do hard things like facing a nightmare and rescripting, participation should always be voluntary. Exposure-based interventions may undermine learning or increase avoidance when they are implemented without sufficient collaboration or perceived control (Craske et al., 2014).

Using the multi-component model of nightmare maintenance as a framework for case conceptualization offers several advantages. The DARC-NESS model supports a collaborative, modular approach in which intervention components are flexibly selected and sequenced based on the child's presentation. This approach also allows clinicians to personalize treatment by targeting the most relevant maintaining processes, promoting clinical efficiency across settings.

## Data Availability

The data analyzed in this study is subject to the following licenses/restrictions: Only a case study was used for demonstration purposes. Requests to access these datasets should be directed to lisa-cromer@utulsa.edu.
